# Recurrent pulmonary cryptococcosis during chronic HBV infection: A case report

**DOI:** 10.1097/MD.0000000000028250

**Published:** 2021-12-17

**Authors:** Huan Liu, Shu Shen, Qiuhui Wang

**Affiliations:** Department of Respiratory, Shenyang Tenth People's Hospital, Shenyang Chest Hospital, Shenyang, China.

**Keywords:** hepatitis B virus, opportunistic infection, pulmonary cryptococcosis, recurrence

## Abstract

**Rationale:**

Pulmonary cryptococcosis is one of the important opportunistic infections and has a wide range of symptoms depending on the underlying conditions. Here, we reported a case living with chronic hepatitis B virus infection who had a recurrent pulmonary cryptococcosis.

**Patient concerns:**

A 51-year-old male patient was admitted to our center because of cough, fatigue, and shortness of breath for 2 weeks.

**Diagnosis:**

Pulmonary infection was suggested by chest computed tomography. Most lab examinations for infection were negative and only cryptococcal antigen testing was positive. Therefore, a clinical diagnosis of pulmonary cryptococcosis was made.

**Interventions:**

Fluconazole (200 mg/day) and bicyclol (50 mg/day) was given orally.

**Outcomes:**

During the follow-up of 3 and 6 months, his conditions improved, and he recovered fully. Moreover, cryptococcal antigen level was 12.57 ng/mL. During the 2-year follow-up, no recurrence occurred.

**Lessons:**

This case highlights the importance of the awareness of opportunistic infections during chronic hepatitis B virus infection, especially the potential of recurrence.

## 1 Introduction

Pulmonary cryptococcosis is an uncommon form of pneumonia that is caused by the inhalation of *Cryptococcus neoformans* and *Cryptococcus gattii* cells. Bird droppings are considered a frequent source of exposure for infection.^[1]^*Cryptococcus* is distributed worldwide and there are no geographical limitations. In a recent study, the annual incidence of cryptococcosis was estimated at 0.03 cases per 100,000 population.^[2]^ In China, pulmonary involvement is present in over half of cryptococcosis infection cases.^[3,4]^ Most patients with pulmonary cryptococcosis are immunocompromised due to various conditions, such as acquired immunodeficiency syndrome (AIDS), immunosuppressive drug use, and hematological malignancies.^[5]^

Due to the more prevalent hepatitis B virus (HBV) infection in immunocompromised humans,^[6,7]^ chronic HBV infection means an immunocompromised condition. HBV infection can impact the clinical presentations, such as lab examinations and outcomes, in patients with cryptococcosis,^[8]^ and this finding unequivocally confirmed the association between HBV and cryptococcosis. Although few reports have investigated cases with co-infection of HBV and cryptococcosis, the precise data regarding patients with co-infection remain unclear. In addition, recurrence of cryptococcosis in a previously asymptomatic patient has not been reported. The patient has provided informed consent for publication of the case.

Here we report our experience of an HBV-infected patient with recurrent pulmonary cryptococcosis who was successfully treated with antifungal therapy. The literature on the co-infection of HBV and cryptococcosis was also reviewed.

## 2 Case presentation

**Figure 1:**
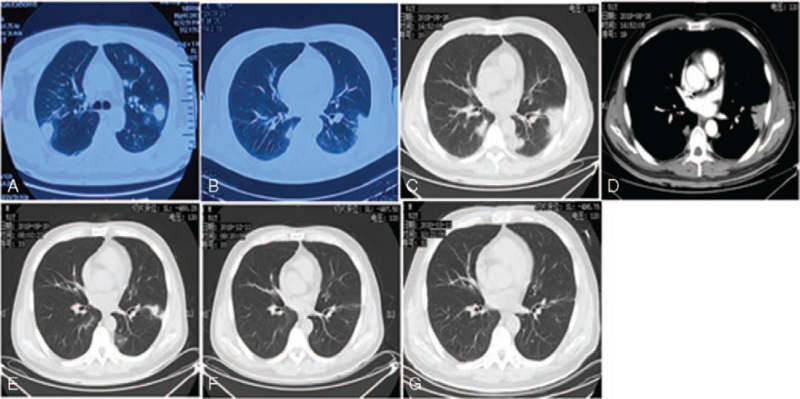
A) Multiple consolidations with blurred margin (before 5 years); B) multiple consolidations and ground-glass shadows (both lungs; on admission); C) multiple consolidations and ground-glass shadows with rough margins, scattering under the pleural surface; D) a maximum CT density of 77 HU revealed by enhanced CT; E) CT findings after 1-month antifungal therapy; F) CT findings after 4-month antifungal therapy; G) CT findings after 6-month antifungal therapy.

A 51-year-old male patient was admitted to our center because of cough, fatigue, and shortness of breath for 2 weeks. He had previously been treated with antibiotics (cephalosporin and azithromycin) over 10 days. However, the symptoms continued. A detailed medical history revealed that he had an episode of pulmonary cryptococcosis 5 years ago (Fig. 1A). He recovered without any treatment and became asymptomatic. He has chronic HBV infection and is undergoing long-term treatment with entecavir. In addition, exposure to bird droppings was reported.

Computed tomography (CT) performed at admission revealed multiple areas of consolidations and ground-glass shadows of bilateral lung fields (Fig. 1B). Chest auscultation demonstrated crude lung respiratory sounds and no evidence of dry and moist rales. His temperature was 36.6°C, respiratory rate was 22 breaths/min, pulse rate was 70 beats/min, and blood pressure was 118/70 mm Hg. Laboratory examinations were notable for C-reactive protein (CRP) of 27.1 mg/L and erythrocyte sedimentation rate (ESR) of 44 mm/h. Other findings such as complete blood cell count, flow cytometry analysis, procalcitonin, *Mycoplasma pneumoniae* (serological assay), HIV, and sputum culture were normal. Blood gas results were as follows: pH 7.426, pO_2_ 67.4 mm Hg, pCO_2_ 39.7 mm Hg, and SpO_2_ 93.4%.

Pulmonary infection was first considered, and piperacillin sodium/tazobactam sodium was given empirically for 1 week. Contrast-enhanced CT was then performed and the results showed multiple consolidations and ground-glass shadows with rough margins and a maximum CT density of 77 HU, with scattering under the pleural surface (Fig. 1C and D). No obvious changes were observed compared with the previous CT scan. For rapid identification of the etiology, bronchoscopic examinations (such as BALF culture and mNGS) were performed. However, no additional information was available. Further examinations were performed; the lab findings included T-SPOT.TB (−), PPD (−), tumor markers (−), p- and c-ANCA (−), (1 → 3)-β-D-glucans (−), and GM assay (−). Notably, cryptococcal antigen testing was positive (55.85 ng/mL; reference value, <8.0 ng/mL). Therefore, a clinical diagnosis of pulmonary cryptococcosis was made.

The patient was orally treated with fluconazole (200 mg/day) and bicyclol (50 mg/day). After 3 weeks of antifungal therapy, his symptoms disappeared and the abnormality on CT scanning had improved (Fig. 1E). During the follow-up of 3 and 6 months, his conditions improved again, and he recovered fully (Fig. 1F and G). Cryptococcal antigen level was 12.57 ng/mL. During the 2-year follow-up, no recurrence occurred.

## 3 Discussion

In this case report, we describe a patient with recurrent cryptococcosis who was living with HBV infection. Pulmonary cryptococcosis, a type of opportunistic fungal infection, is caused by encapsulated *C gattii* and *C neoformans* fungi that are frequently found in bird droppings. Pulmonary cryptococcosis has become an emerging disease in immunocompromised and immunocompetent patients.

HBV infection can lead to a chronic inflammation of the liver and is the most common etiology of liver cirrhosis and cancer.^[9]^ Cryptococcosis may be associated with a status of altered host defense responses, and most cases (over 90%) are associated with AIDS.^[10]^ Patients with liver disease have an increased susceptibility to infections that are often secondary to impaired phagocytic function. For example, hepatic cirrhosis is a disease that leads to immunodeficiency.^[11]^ Hence, the infection increases the risk of opportunistic infections, such as cryptosporidium infection.^[12]^ Furthermore, compared with patients without opportunistic infections, cirrhosis patients with opportunistic infections have lower levels of CD8+ T cells and NK cells.^[13]^ The reduced host immune response partially contributes to the progression of HBV infection and opportunistic infections.^[14]^ In addition, decreased complement levels, corticosteroid use, antibiotics use, and invasive procedures may significantly increase the risk of cryptococcosis.^[15,16]^ Although cryptococcosis patients living with HBV do not have specific symptoms, once cryptococcosis affects the brain, a poor outcome is expected.^[17]^ Therefore, physicians must be aware of pulmonary cryptococcosis in patients living with HBV, especially the disseminated type of cryptococcosis.

Several reasons may explain the recurrence of the current patient's pulmonary cryptococcosis, such as chronic HBV infection and poor adherence and compliance. Although no therapy was given when the patient first fell ill with cryptococcosis and the individual was asymptomatic, *Cryptococcus* remains colonized in the tracheobronchial tree in infected individuals.^[18]^ However, chronic HBV infection is associated with an altered immune response, which contributes to the increased risk of pulmonary cryptococcosis. Other causes contributing to the recurrence of pulmonary cryptococcosis include immunocompromised status, suboptimal therapy, and economic challenges.^[19,20]^

The precise diagnosis of a disease is paramount to successful treatment. Without an accurate diagnosis, an appropriate treatment cannot be initiated. The presentation of pulmonary cryptococcosis varies widely, ranging from asymptomatic colonization to life-threatening pneumonia. The most common symptoms include cough, expectoration, and fever. Because of the nonspecific manifestations, diagnostic delay or misdiagnosis is a concern. Previous studies detailed the CT features of pulmonary cryptococcosis.^[21,22]^ The radiological findings included nodules or mass, multiple nodules, lobular, segmental, or subsegmental consolidations, and bronchopneumonia.^[22–24]^ Although consolidations and ground-glass shadows were also observed in this case bilaterally, an accurate diagnosis could not be made. Microbiological methods are useful for the diagnosis of pulmonary cryptococcosis.^[25,26]^ However, in this case, the sputum culture and smear results were negative. In addition, the patient refused invasive operation. Thus, the histological method was not used in this case. This case was eventually successfully diagnosed by a cryptococcal antigen test.^[27]^

This case is of particular interest, as it not only provides further evidence that patients infected with cryptococcal cells have variable symptoms ranging from asymptomatic to nonspecific symptoms including cough, sputum, and chest pain. Due to immune responses altered by HBV, opportunistic infections may be easily encountered. The diagnosis of cryptococcosis remains difficult and further research should be performed to improve the diagnostic accuracy. In addition, awareness of fungal disease should not be limited only to AIDS cases, but also to patients with chronic infections. These measures should be incorporated into routine clinical practice, especially in areas with high cryptococcosis burden.

## Author contributions

**Conceptualization:** Shu Shen.

**Data curation:** Qiuhui Wang.

**Project administration:** Huan Liu.

**Validation:** Shu Shen.

**Writing – original draft:** Huan Liu.

**Writing – review & editing:** Huan Liu.
